# State-of-the-Art Fusion-Finder Algorithms Sensitivity and Specificity

**DOI:** 10.1155/2013/340620

**Published:** 2013-02-17

**Authors:** Matteo Carrara, Marco Beccuti, Fulvio Lazzarato, Federica Cavallo, Francesca Cordero, Susanna Donatelli, Raffaele A. Calogero

**Affiliations:** ^1^Department of Molecular Biotechnology and Health Sciences, University of Torino, Via Nizza 52, 10126 Torino, Italy; ^2^Department of Computer Science, University of Torino, C.So Svizzera 185, 10149 Torino, Italy; ^3^Unit of Cancer Epidemiology, Department of Biomedical Sciences and Human Oncology, University of Torino, 10126 Torino, Italy

## Abstract

*Background*. Gene fusions arising from chromosomal translocations have been implicated in cancer. RNA-seq has the potential to discover such rearrangements generating functional proteins (chimera/fusion). Recently, many methods for chimeras detection have been published. However, specificity and sensitivity of those tools were not extensively investigated in a comparative way. *Results*. We tested eight fusion-detection tools (FusionHunter, FusionMap, FusionFinder, MapSplice, deFuse, Bellerophontes, ChimeraScan, and TopHat-fusion) to detect fusion events using synthetic and real datasets encompassing chimeras. The comparison analysis run only on synthetic data could generate misleading results since we found no counterpart on real dataset. Furthermore, most tools report a very high number of false positive chimeras. In particular, the most sensitive tool, ChimeraScan, reports a large number of false positives that we were able to significantly reduce by devising and applying two filters to remove fusions not supported by fusion junction-spanning reads or encompassing large intronic regions. *Conclusions*. The discordant results obtained using synthetic and real datasets suggest that synthetic datasets encompassing fusion events may not fully catch the complexity of RNA-seq experiment. Moreover, fusion detection tools are still limited in sensitivity or specificity; thus, there is space for further improvement in the fusion-finder algorithms.

## 1. Background

Direct sequencing of messenger RNA transcripts using the RNA-seq protocol [1] is rapidly becoming the standard method for detecting and quantifying genes being expressed in a cell. One of the key features observed when analyzing cancer genomes is chromosomal abnormality. Genome rearrangements could result in aberrant fusion genes, and a number of them have been found to play important roles in carcinogenesis [2]. The discovery of novel gene fusions can lead to a better comprehension of cancer progression and development. The emergence of deep sequencing of transcriptome, known as RNA-seq, has opened many opportunities for the identification of this class of genomic alterations, leading to the discovery of novel chimeric transcripts in many cancers [2]. In this paper, we compare eight fusion-finder softwares to evaluate their relative efficacy in detecting in fusion events.

## 2. Results

### 2.1. Fusion Finders

At the best of our knowledge, we have identified Bellerophontes [3], ChimeraScan [4], deFuse [5], FusionFinder [6], FusionHunter [7], FusionMap [8], MapSplice [9], and TopHat-fusion [10] as the most used tools for chimeras detection.

The tools can be organized in various subgroups on the basis of their alignment strategies. In this paper, we propose the following classification: *Whole paired-end, Paired-end + fragmentation*, and* Direct fragmentation.*

In the *Whole paired-end* approach, tools align the full-length paired-end reads on a reference and use discordant alignments to generate a set of putative fusion events which are finally selected using several additional pieces of information or filtering steps.

Instead, the tools in the *Paired-end + fragmentation* class derive the putative fusion events in two steps. First, as in the *Whole paired-end* approach, the full-length paired-end reads are aligned on a reference, and the discordant alignments are used to generate new pseudoreference including only the identified putative fusion events. Then, reads unaligned in the first step are fragmented and realigned on the pseudoreference to identify junction-spanning reads. Only the putative fusion events associated with junction-spanning reads are selected as input to the filtering step.

Finally, the tools based on *Direct fragmentation* do not directly exploit paired-end information; they fragment every read before the first alignment and find fusion candidates aligning those fragments to a genomic reference.

The putative fusion events are then selected implementing a set of filtering steps. 

According to the previous classification the eight tools compared in this paper can be grouped as following:

1. deFuse and FusionHunter are *Whole Paired-end *based tools;
2. TopHat-fusion, ChimeraScan, and Bellerophontes are *Paired-end + fragmentation* based tools;
3. MapSplice, FusionMap, and FusionFinder are *Direct fragmentation* based tools.

Since all the considered tools implement a set of filters to reduce the number of false positive fusion events, a brief description of these filters is reported.

*Paired-End Information Filter.* It uses the distance between the tags of a pair to validate the alignment on a fusion.

*Anchor Length Filter.* Anchor length is an important metric for quality evaluation of a read spanning across a fusion junction, and it is defined as the number of nucleotides overlapping each side of the break point. The filter removes all the junction-spanning reads having the anchor length lower than a threshold.

*Read-Through Transcripts Filter.* It removes the RNA molecules formed by exons of adjacent genes, usually generated by the RNA-polymerase failing the recognition of the gene end. 

*Junction-Spanning Reads Filter.* It discards all fusion events supported by a number of junction-spanning reads lower than a threshold.

*PCR-Artifact Filter.* It identifies and removes all duplicated reads introduced by the PCR amplification process.

*Homology-Based Filter.* It removes candidate fusions with a high number of reads on homologous or repetitive regions.

*Quality-Based Filters.* It is a group of filters that uses different metrics (e.g., entropy, base quality, etc.) for computing the fusion quality. Then, all the candidates with quality lower than a threshold are removed.

In Table 1, we report the implemented filters for each considered tool.

### 2.2. Fusion Detection Sensitivity

To compare the sensitivity of chimera finder algorithms, we used three datasets.

The first dataset is synthetic (*FM_set*), while the other two are based on real data (*Edgren_set* [11] and *Berger_set* [12]). All the datasets are paired-end ones. The synthetic set is composed of 75 nts reads, while the other two contain 50 nts reads. *FM_set* encompasses 50 fusion events, supported by 9 to 8852 paired-end reads. The *Edgren_set* encompasses a total of 27 experimentally validated fusion genes, detected in BT-474, SK-BR-3, KPL-4, and MCF-7 breast cancer cell lines [11]. *Berger_set* encompasses a total of 12 experimentally validated fusion genes, detected in 501 Mel (Melanoma), K-562 (Leukemia) cell lines, and in 5 samples from primary human melanoma [12].

In this analysis of sensitivity, we considered three parameters: (i) the total number of true positive fusions detected by the different tools (called *all*), (ii) the number of true positive fusions detected by the correct orientation of the two genes (called *right*), and (iii) the number of true positive fusions detected by erroneous orientation of the two genes (called *wrong*).

**Figure 1:**
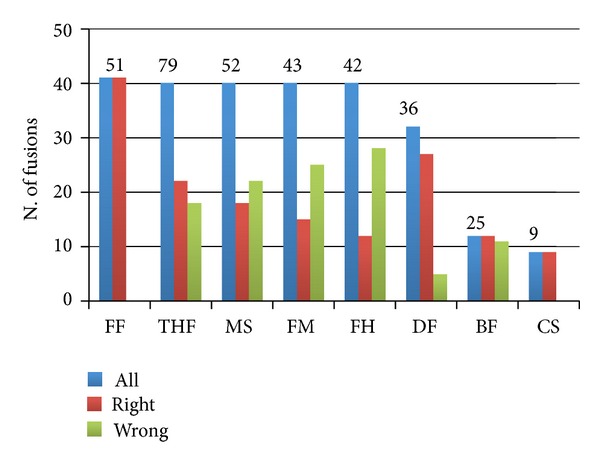
*Fusion events detection performances on positive data set encompassing 50 synthetic fusion events* (*FM_set*). Total number of detected fusions is shown on the top of each bar set. FF: FusionFinder; THF: TopHat-fusion; MS: MapSplice; FM: FusionMap; FH: FusionHunter; DF: defuse; BF: Bellerophontes; CS: ChimeraScan.

Using the synthetic *FM_set*, five out of eight analyzed tools show a good sensitivity, since they detect 40 out of 50 fusions (Figure 1(a), blue bars). ChimeraScan was the least sensitive detecting only nine out of 50 fusions (Figure 1). FusionFinder and ChimeraScan were the only tools that did not make any error in the detection of the fusions orientation (Figure 1(a), red bars). It is notable that Bellerophontes was calling all fusion events in both possible orientations, essentially leaving to further down-stream analysis the definition of the correct orientation of fusion events.

**Figure 2:**
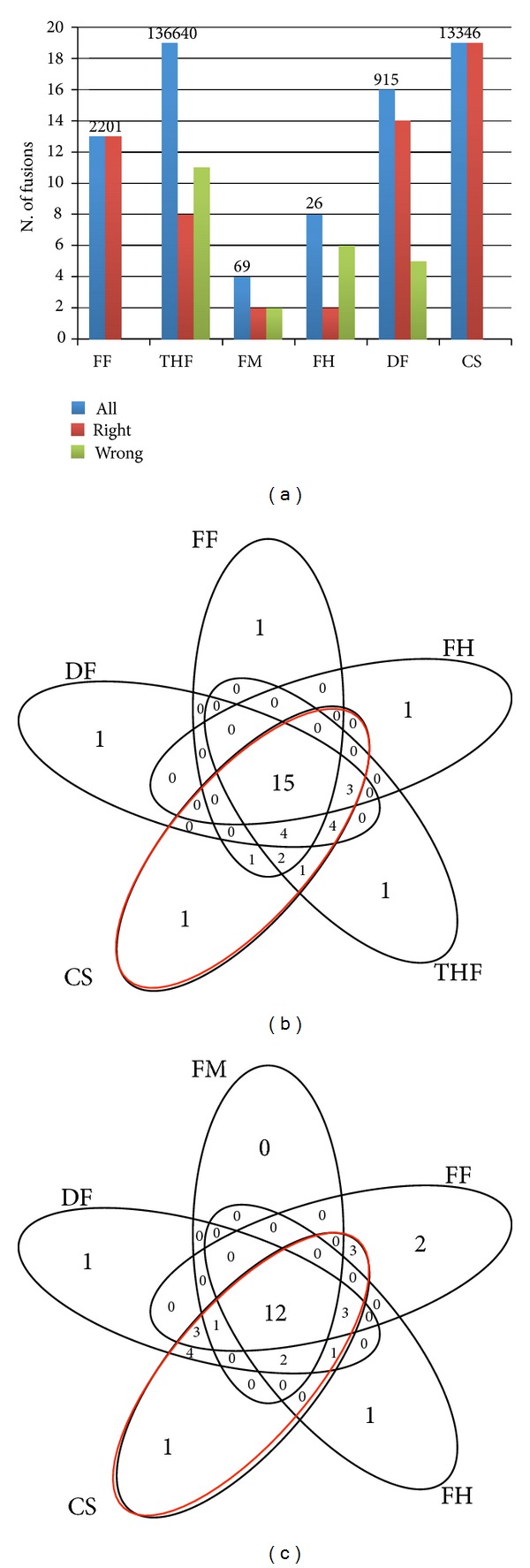
*Analysis of sensitivity of fusion finders in a real data set encompassing 27 validated fusions* (*Edgren_set*). (a) Total number of detected fusions is shown on the top of each bar set. (b) and (c) Venn diagrams showing the overlaps between fusions founded by different tools. The ellipse of the ChimeraScan is highlighted in red. FF: FusionFinder; THF: TopHat-fusion; MS: MapSplice; FM: FusionMap; FH:FusionHunter; DF: defuse; BF: Bellerophontes; CS: ChimeraScan.

The same analysis performed on the *Edgren_set* provided a completely different view of sensitivity of the analyzed tools (Figure 2). From this analysis, ChimeraScan performed better than all the other tools concerning the number of detected chimeras and the correct orientation of the fusion events; 19 out of 27 fusions were all detected in the right orientation. TopHat-fusion was as sensitive as ChimeraScan detecting 19 chimeras, but only 8 of them were in the correct orientation. Furthermore, the 19 true fusions were part of a set of more than 130000 events, which makes quite difficult the task of purging the false positives. deFuse and FusionFinder came in sensitivity after ChimeraScan and TopHat-fusion, detecting 16 and 13 chimeras, respectively. FusionHunter and FusionMap performance was very poor, with 8 and 4 detected chimeras. MapSplice and Bellerophontes data could not be collected because, after 10 days from the beginning of the analysis, the tools were still filtering fusions events.

We also evaluated the level of overlap between the various tools for the *Edgren_set* chimeras (Figures 2(b) and 2(c)). ChimeraScan encompasses all genes detected by FusionMap and the majority of the fusions detected by the other tools.

Another interesting point is the strong difference between tools in the number of fusions called. At the two extremes are TopHat-fusion, calling more than 130000 chimeras, and FusionHunter, calling only 26 chimeras. We also observed that the best two tools, ChimeraScan and TopHat-fusion, are the ones with the highest number of called fusions. The number of called chimeras is, however, not proportional with the number of detected true positives; for example, both ChimeraScan and TopHat-fusion detect 19 true positives. However, the number of chimeras detected by TopHat-fusion is approximately ten times greater than those detected by ChimeraScan (Figure 2).

**Figure 3:**
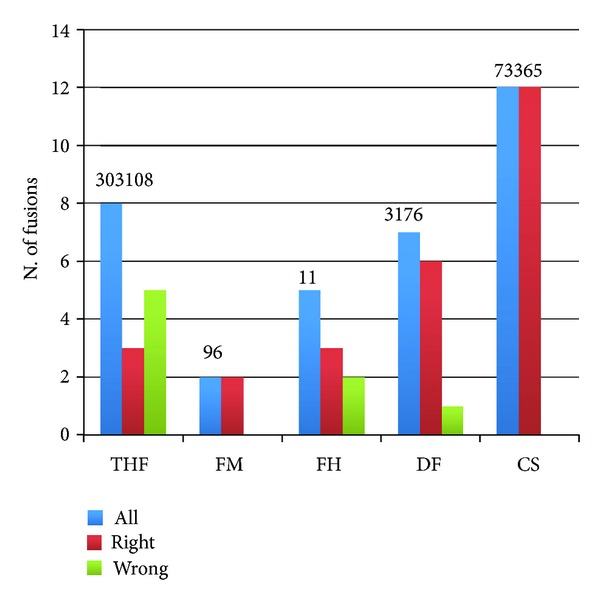
*Analysis of sensitivity of fusion finders on a real data set encompassing 12 validated fusions* (*Berger_set*). Total number of detected fusions is shown on the top of each set of bars. FM: FusionMap; FH: FusionHunter; DF: defuse; CS: ChimeraScan; THF: TopHat-fusion.

We further confirm that ChimeraScan performs better than the other tools also on *Berger_set* (Figure 3). It is notable that the fusion discovery sensitivities for FusionMap, FusionHunter, deFuse, TopHat-fusion, and ChimeraScan, previously observed in the *Edgren_set*, are also kept in the *Berger_set*, and TopHat-fusion is again the best tool in sensitivity after ChimeraScan. However, it is notable that also in *Berger_set*, we have a very high number of called fusions for ChimeraScan and TopHat-fusion, which may make their use in a real experimental setting unpractical.

### 2.3. False Discovery of Fusions

As shown in the previous paragraph, real datasets are useful to test tools in conditions that resemble their everyday usage. However, real datasets have the limitation that the exact number of true positive fusions is not known; thus, false positive detection cannot be assessed. For this reason, we have used a negative data set (called *negative_set*) encompassing 70 million reads 2 × 50 nts [13].

**Figure 4:**
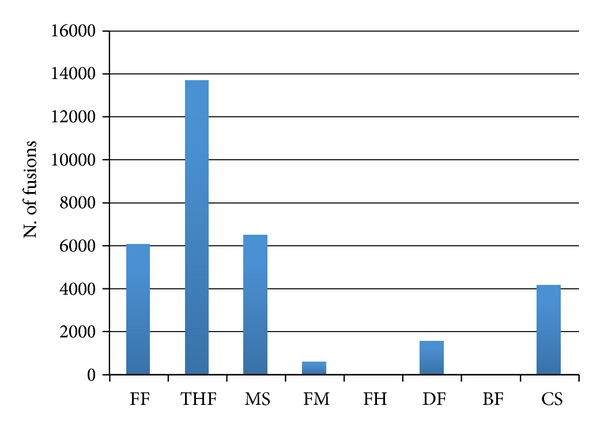
*False positive fusion detected using a synthetic dataset without chimeras*. FF: FusionFinder; THF: TopHat-fusion; MS: MapSplice; FM: FusionMap; FH: FusionHunter; DF: defuse; BF: Bellerophontes; CS: ChimeraScan.

FusionHunter and Bellerophontes are the only tools not detecting false chimeras in the negative dataset (Figure 4). The number of false positives increases progressively from FusionMap, deFuse, ChimeraScan, FusionFinder, and MapsSplice to TopHat-fusion, which has the highest number of false positive detected chimeras.

We try to evaluate, for ChimeraScan, if there is a bias in the discovery approach of the tool, which could lead to find the same fusions in different datasets. Intersecting the fusions detected in the *Edgren_set* and in the *Berger_set* and those detected in the *negative_set,* the overlap is marginal. Sixty fusions are found in common between the *negative_set* and the *Edgren_set*, 197 between the *negative_set* and the *Berger_set,* and only 38 fusions are in common between the previous two comparisons. This observation suggests that false positives are mainly dataset specific and not significantly biased by the tool characteristics.

### 2.4. Optimizing Removal of False Positive Fusions

Being ChimeraScan the most efficient tool in detection of fusion events in the right orientation, we evaluated various filtering approaches to reduce the false positive fusions contaminating the real fusion events. Specifically, we used the characteristics of the chimeras detected in the *negative_set* to define false positive filters. We observed that many chimeras found in the negative set by ChimeraScan were supported by reads encompassing the two exons of the genes involved in the fusion and were missing reads spanning over the fusion junction. Since the presence of junction-spanning reads is an important positive parameter for the definition of a fusion event [2], we decided to filter-out all the chimeras detected in the *Edgren_set* but not supported by reads spanning over the fusion junction. The filter is very efficient since we retain only 681 fusions out of the initial 13346 detected by ChimeraScan. Concerning the true positives, 17 out of the 19 detected fusions are also retained. RPS6KB1:SNF8 and CPNE1:PI3 are instead lost.

It is interesting to note that RPS6KB1:SNF8 can be detected by deFuse, FusionHunter, and TopHat-fusion, while CPNE1:PI3 could be found by FusionFinder and TopHat-fusion. All the previously mentioned methods manage to detect spanning reads for RPS6KB1:SNF8 and CPNE1:PI3, suggesting that ChimeraScan algorithm fails to detect those spanning reads. We are currently trying to find out the reason why ChimeraScan failed in detecting the prviously mentioned fusion junction spanning reads. Furthermore, tools already implementing a filter based on the number of junction-spanning reads consistently show a lower number of reported fusions.

We have also observed the presence of a high number of fusions encompassing intronic region in the fusions detected in *negative_set*. These fusions generate very large transcripts, which do not produce in frame transcripts. Removing them from the 681 fusions detected in the *Edgren_set,* we retain 249 chimeras, without loss of true positives. Again, some of tools include alignment approaches with an effect similar to this filter by aligning reads to the transcriptome.

Although 249 chimeras represent a significant reduction of the initial number of detected chimeras, they are still too many to be all experimentally validated. Sorting the 249 chimeras in descending order, on the basis of the number of fusion junction-spanning reads, we show that with the top 17 chimeras, 10 were part of the 17 true positives. The rationale of this ranking procedure is that biological effect also depends on the amount of the expressed mRNA; thus, highly expressed fusions, that is, fusions with a high number of junction-spanning reads, might have a more important role in cancer physiology.

## 3. Discussion 

The main goal of this paper is to understand strength and limits of the main fusion detection software currently available. To reach our aim, we have evaluated sensitivity and false fusion discovery for eight state-of-the-art fusion finders: Bellerophontes, FusionHunter, FusionMap, FusionFinder, MapSplice, deFuse, ChimeraScan, and TopHat-fusion. We run this comparison using both synthetic and real datasets.

Concerning sensitivity, we observed that a comparison analysis run only on synthetic data could generate misleading results. Sensitivity analysis run on the synthetic data only results in ChimeraScan being the least sensitive tool, while it is actually the most sensitive tool on real datasets. We think that discrepancies between results obtained on synthetic and real data are due to the actual lack of knowledge of the real complexity of RNA-seq data that does not allow the construction of fully significant synthetic datasets. The analysis of real datasets allows us to identify ChimeraScan as the most sensitive tool for chimeras detection although ChimeraScan output is affected by a very high number of called fusions, a number too big to make a functional experimental validation feasible. A synthetic dataset, free of fusion events by construction (*negative_set*), represents an interesting instrument to understand the basic characteristics of false fusions detected by ChimeraScan and to define specific filters to remove them. It was observed that the main characteristics of false positive fusions in the *negative_set* are both the lack of fusion junction-spanning reads and the inclusion of intronic regions in the fusion. The application of two filters, based on the previous false positive characteristics, proves to be very efficient in reducing the 13346 initially detected fusions (*Edgren_set*) to 249, with the limited loss of two true positive chimeras. It is also notable that ranking chimeras on the basis of the number of fusion junction-supporting reads also helps to further narrow the set of chimera to be experimentally validated.

## 4. Conclusions 

This paper highlights that fusion detection tools are still not fully adequate to provide a direct solution for the discovery of chimeras in a dataset. Many algorithms have been proposed, and each of them has specific biases at the level of sensitivity or specificity. Tools having low sensitivity are also characterized by a limited number of false positives. Moreover, results obtained by the low sensitivity tools show very limited overlap in the results. On the other hand, tools as ChimeraScan and TopHat-fusion show a good sensitivity but also the presence of a high number of false positives. Filters devoted to the removal of false positives can significantly improve the ratio between true positives and false positives, but there is clearly space for algorithm improvements.

## 5. Methods

### 5.1. Fusion Detection Softwares and Data Analysis

FusionHunter, FusionMap, FusionFinder, MapSplice, deFuse, ChimeraScan, Bellerophontes, and TopHat-fusion were downloaded from the repositories indicated in their publications and installed following requirements indicated in their manuals. Software was run using default configuration. All analyses were performed on a 48-core AMD server with 512 Gb RAM and 9 Tb HD, running linux SUSE Enterprise 11. Statistics and data parsing were executed using R scripting, taking advantage of Bioconductor [14] packages, that is, Biostrings, org.Hs.eg.db, GenomicRanges, and oneChannelGUI [15].

### 5.2. Positive Dataset

FusionMap developers provide a synthetic dataset of simulated paired-end RNA-seq reads (~60,000 pairs of reads, 75 nt, fragment size = 158 bp). Fifty fusions are represented with a range of supporting pairs going from 9 to 8852. Real datasets encompassing experimentally validated chimeras were retrieved from NCBI Sequence Read Archive (SRA:SRP003186) as described in [11] and from NCBI Gene Expression Omnibus (http://www.ncbi.nlm.nih.gov/geo/), SuperSeries Accession no. GSE17593 as described in [12].

### 5.3. Negative Dataset

The negative dataset was generated using BEERS [16], and its construction is described in [13]. 

## Figures and Tables

**Table 1:** Filtering steps embedded in the algorithms.

Filters	Fusion finders							
	FF	THF	MS	FM	FH	DF	BF	CS
Pair distance	X					X	X	X
Anchor length		X			X			X
Read-through	X	X		X	X		X	
Junction-spanning				X	X		X	
PCR artifact				X	X		X	
Homology	X	X					X	
Quality			X	X				

FF: FusionFinder; THF: TopHat-fusion; MS: MapSplice; FM: FusionMap; FH: FusionHunter; DF: deFuse; BF: Bellerophontes; CS: ChimeraScan.
